# Optimizing Desolvation Conditions for Glutathione-Cross-Linked Bovine Serum Albumin Nanoparticles: Implication for Intravenous Drug Delivery

**DOI:** 10.7759/cureus.69514

**Published:** 2024-09-16

**Authors:** Hamid J Hasan, Mowafaq M Ghareeb

**Affiliations:** 1 Department of Pharmaceutics, College of Pharmacy, University of Baghdad, Oncology Teaching Hospital - Medical City, Baghdad, IRQ; 2 Department of Pharmaceutics, College of Pharmacy, University of Baghdad, Baghdad, IRQ

**Keywords:** bovine serum albumin nanoparticles, desolvation method, glutathione, hansen solubility parameters, organic solvents

## Abstract

Introduction: Protein-based nanocarriers, particularly albumin nanoparticles (NPs), offer numerous advantages when compared to other nanomaterials. These carriers are characterized by biocompatibility, biodegradability, reduced immunogenicity, and decreased cytotoxicity. Moreover, proteins possess an inherent ability to target tumor cells directly or indirectly.

Aim: This study aims to investigate the impact of various organic solvents on the characteristics of synthesized bovine serum albumin NPs (BSA NPs).

Method: BSA NPs were produced using methanol, acetone, ethanol, dimethylsulfoxide (DMSO), and acetonitrile through the desolvation technique to achieve particles of acceptable size. Dynamic light scattering (DLS), blood compatibility assays, polyacrylamide gel electrophoresis (PAGE), and size exclusion chromatography (SEC) were employed to elucidate the properties of the generated NPs. The cytotoxicity of BSA NPs prepared under different conditions was assessed using Michigan Cancer Foundation - Mammary Adenocarcinoma - Breast Cancer 231 cells (MDA-MB-231 cells).

Results: The particle size of the synthesized NPs varied based on the organic solvent utilized, with the smallest size of 114.2 nm observed with methanol. Blood compatibility results indicated no abnormal interactions between BSA NPs and blood components. PAGE analysis revealed a strong band near 72 kDa for untreated BSA and BSA treated with all organic solvents. In SEC, the retention time of native albumin was 6.65 min, while the average retention times of the prepared BSA NPs ranged from 5.14 to 5.21 min, showing similarity to the native protein. Except for NPs produced with methanol and acetonitrile, cytotoxicity testing on MDA-MB-231 cells demonstrated no significant harmful effects at various concentrations (0-500 μg/mL).

Conclusion: The choice of desolvating agent significantly influences the size of BSA NPs. Various factors, such as solvent characteristics like hydrogen bonds, polarity, dielectric constant, and functional groups, can affect the particle size and structure of BSA NPs. The compatibility of cross-linked BSA NPs with blood components suggests their potential for intravenous drug delivery applications.

## Introduction

Drug and bioactive agent distribution that is site-specific or time-controlled can be effectively achieved using nanoparticles (NPs). The focus of pharmaceutical nanotechnology is on the formulation of drugs in biocompatible nanoforms, which can improve drug delivery. NPs improve medication stability, lengthen the duration of the drug's impact on the target tissue, improve bioavailability, and enable targeted drug administration, among other ways that pharmaceuticals are made more effective and safe [1-3].

Bovine serum albumin (BSA) is a popular macromolecular carrier because of its non-toxic, non-immunogenic, biocompatible, and biodegradable qualities. Through transcytosis mediated by the gp60 receptor, it was able to accumulate in tumors. Usually, modifications are made to the surface carboxylic and amino groups. The great loading capacity of albumin-based NPs and their well-tolerated nature, free of major side effects, have drawn a lot of interest to them recently as potential drug delivery vehicles [4].

One popular and straightforward process for creating albumin NPs is the desolvation or coacervation procedure [5]. This approach involves adding a desolvating chemical, for example, ethanol or acetone, to an albumin aqueous solution while continuously swirling the mixture with a magnet until turbidity is seen in the solution. To obtain albumin NPs of desirable size, two crucial parameters are the rate of flow and the amount of the additional desolvating agent. Following the desolvating agent addition, a cross-linker, such as a glutathione solution, is needed to stabilize the unstable particles. The prepared suspension must be continuously stirred for three hours in order to fully crosslink all of the protein's amino acid residues [6].

The current study's goal is to find out how various desolvating agents influence the particle size and properties of BSA NPs that are made using the desolvation process. Functional groups, dielectric constants, hydrogen bonding potentials, and polarity vary between the desolvating agents. On breast cancer cells, MDA-MB-231 cells, the cytotoxic effects of BSA NPs of varying sizes, synthesized using different desolvating agents, were assessed.

## Materials and methods

Materials

BSA and L-glutathione reduced were supplied by Hangzhou Hyper Chemicals Limited, China. Absolute ethanol and acetonitrile were obtained from Merck KGaA (Darmstadt, Germany). Acetone for HPLC-isocratic grade was obtained from CARLO ERBA Reagents GmbH (Emmendingen, Germany), methanol for HPLC was obtained from HiMedia Laboratories Private Limited (Mumbai, India), and DMSO was obtained from Loba Chemie Pvt. Ltd. (Mumbai, India). Coomasie brilliant blue R-250 was supplied by HIMEDA. Modified eagle medium (DMEM) was supplied by Giboco (New York, USA) and MTT (3-(4,5-dimethylthiazol-2-yl)2,5-diphenyl tetrazolium bromide) from Promega Corporation (Madison, Wisconsin).

Methods

Preparation of BSA NPs

Preparation of NPs was achieved using a formerly defined desolvation method [7,8]. Acetone, methanol, ethanol, DMSO, and acetonitrile were used separately as de-solvating agents. Additionally, 2 g of BSA was dissolved in 100 mL of deionized water. NPs were created by drop-wise addition of organic solvents to 5 mL of BSA solution at a flow rate of 1.0 mL/min using a syringe pump (Beijing KellyMed Co. Ltd., China) with constant stirring (1,250 rpm) at room temperature (25 °C) till the solution converted to turbid. After that, 240 μL of 2% glutathione solution was then added to bring crosslinking of NPs, and the solution was stirred for an extra three hours. Dialysis involves placing the NP's suspension in a dialysis membrane with a molecular weight cutoff of 10,000 Da that allows the desolvent molecules to diffuse out while retaining the NPs. Then, the desolvating agent, free albumin, and unreacted glutathione were removed by two rounds of centrifugations at 15,000 rpm for up to 15 min. The pellets at the bottom of the centrifuge tubes were collected by dispersing in 10 mL of deionized water [9]. Multiple washing steps with deionized water to rinse out any remaining de-solvent agent from the NPs suspension.

Physicochemical Characterization of NPs

Dynamic light scattering (DLS) by using a zeta-sizer device (Malvern, Panalytical, UK) revealed the physicochemical properties (particle size, PDI, and zeta potential) of the produced NPs. By maintaining the temperature of 25 °C and a scattering angle of 90°, this apparatus is able to estimate the intensity of light that was scattered by the particles in the experienced sample as a time meaning [10,11].

Solvent Properties

Table 1 lists the desolvating compounds' solubility parameters, dielectric constant, and dipole moment. The total, sometimes known as the Hildebrand solubility parameter, is equal to the sum of squares of Hansen solubility parameters (HSP). These parameters result from (molecular) permanent dipole-permanent dipole forces, (molecular) hydrogen bonding (electron exchange), and (atomic) dispersion forces. Similar HSP materials exhibit strong affine attraction for one another. The degree of the interaction in a specific circumstance depends on the degree of resemblance in that situation. Polar protic solvents with O-H bonds and capable participation in hydrogen bonding are water, ethanol, and methanol. Acetone and acetonitrile, which are polar aprotic solvents, do not engage in hydrogen bonding in meanwhile. The dielectric constants of an aqueous solution are found from the dielectric constants of each of its components and their relative volume by means of the mixture equation of Silberstein:

$&epsilon; = &epsilon;1 ῡ1 + &epsilon;2 ῡ2$

Where

ε represents the dielectric constant of the mixture and denotes the dielectric constant of every component and ῡ1 and ῡ2 refer to their relative volumes, respectively [12].

**Table 1:** Solubility parameters of organic solvents ^a^Hydrogen bonding solubility parameter (Mpa0.5) ^b^Polar solubility parameter (Mpa0.5) ^c^Dispersion solubility parameter (Mpa0.5) ^d^Total (Hildebrand) solubility parameter (Mpa0.5) (δ2= δ2 D+δ2 H+δ2 P)

Solvent	Dielectric constant (μ)	Dipole moment	δ _H_^a^	δ _P_^b^	δ _D_^c^	δ ^d^
acetone	21.00	2.88	7.00	10.40	15.5	19.90
Ethanol	24.50	1.69	19.40	8.80	15.8	26.20
methanol	33.00	1.70	22.30	12.30	14.7	26.90
acetonitrile	37.50	3.94	6.10	18.00	15.3	24.30
DMSO	46.68	3.96	10.20	16.40	18.4	26.70
water	88.00	1.84	42.30	16.00	15.5	48.00

Structural Analysis of BSA

Protein aggregates are generally characterized using sodium dodecyl sulfate-polyacrylamide gel electrophoresis (SDS-PAGE) and size-exclusion liquid chromatography (SEC-HPLC) [13]. In the case of SDS-PAGE, the procedure includes mixing samples with the sample buffer solution, loading them into a 10% polyacrylamide gel, and then detecting by staining using Coomassie Blue R-250. The gel image was recorded using a Bio-Rad Molecular Imager (ChemiDoc XRS+ Imaging System, France) [14].

Analytical SEC is achieved to evaluate the quality of the sample or to check the properties of a biomolecule. It is used to evaluate protein stability, study complex formation, and evaluate the tendency to aggregate and the quantity of aggregates [15]. Analysis for pure BSA and BSA NPs was performed by SEC or gel filtration chromatography using a TSK-GEL® G3000SW (Tosoh Bioscience, Germany) with (7.8 mm internal diameter and 30 cm length). The column was connected to an HPLC device (Shimadzu, Japan) equipped with a DGU-20A degasser, an LC-20AD pump, SPD-20A UV-visible detector, and a LabSolutions data acquisition program. The temperature was fixed at 25 °C. The mobile phase used was ultra-pure Milli-Q water (distilled water purified with ultrapure filtration apparatus) at a flow rate of 1.0 mL/min. Detection was carried out at 214 nm. An aliquot of 50 µL of aqueous solutions of the native protein and NPs prepared with the different organic solvents was injected into the SEC system [16].

Human Blood Compatibility

Hemolysis test: The blood was kindly supplied by the Clinic of Oncology Teaching Hospital, Directorate of Medical City (Baghdad, Iraq). The red blood cells (RBCs) were prepared for hemolytic assay depending on the reported technique [17]. Additionally, 2 mL of fresh blood of a male human was drawn from a willing, healthy man, stabilized with ethylenediaminetetraacetic acid (EDTA), and centrifuged at 2,000 rpm for 10 min to eliminate the plasma as supernatant. The resulting precipitation containing RBCs was purified two times by successive washing with phosphate-buffered saline (PBS, pH 7.4). After 10 times dilution of 2 mL of the suspension of RBC with PBS, 200 µL of RBC suspension separately was added to 800 µL of all NPs samples, followed by mild shaking and incubating for up to two hours at room temperature using a incubator (Memmert, Germany). By the same procedure, negative and positive controls were prepared by the addition of 200 µL of RBC suspension distinctly to 800 µL of PBS and Triton X100 (2% v/v), respectively. Lastly, after centrifugation of all the samples and controls at 10,000 rpm for one minute, the hemoglobin absorbance in supernatants was detected by UV-visible spectrophotometer at 541 nm. In order to determine the hemolytic percentages of the different samples, the absorbance difference between the samples and negative control was divided by that between the positive and negative controls by using the following formula:

Hemolytic activity % = [ABS sample - ABS-ve ctrl]/[ABS +ve ctrl −ABS-ve ctrl ] × 100

The accepted value should be less or equal to 5 [17].

Erythrocyte aggregation test: An aggregation assay was achieved to check if the NPs have a toxic effect on RBCs. The human blood was kindly provided by the Clinic of College of Pharmacy, University of Mustansiriyah (Baghdad, Iraq) and collected in a sterile tube with (EDTA). Centrifugation of about 2 mL of blood was done at 2,000 rpm for 30 minutes in order to separate RBCs. The rinsing of RBC cells was done with PBS two times and diluted 10 times with the same buffer. To evaluate the toxic effect of BSA NPs prepared with the different organic solvents, 100 µL of RBCs was treated with 400 µL of each sample of NPs (in the concentration of 500 µg/mL) dissolved in PBS and incubated for one hour at 37 °C. Erythrocytes were observed at 40 magnifications using a compound microscope connected to a computer that shows the pictures (Optika, Italy) [18].

Cell Viability Study

**Figure 1:**
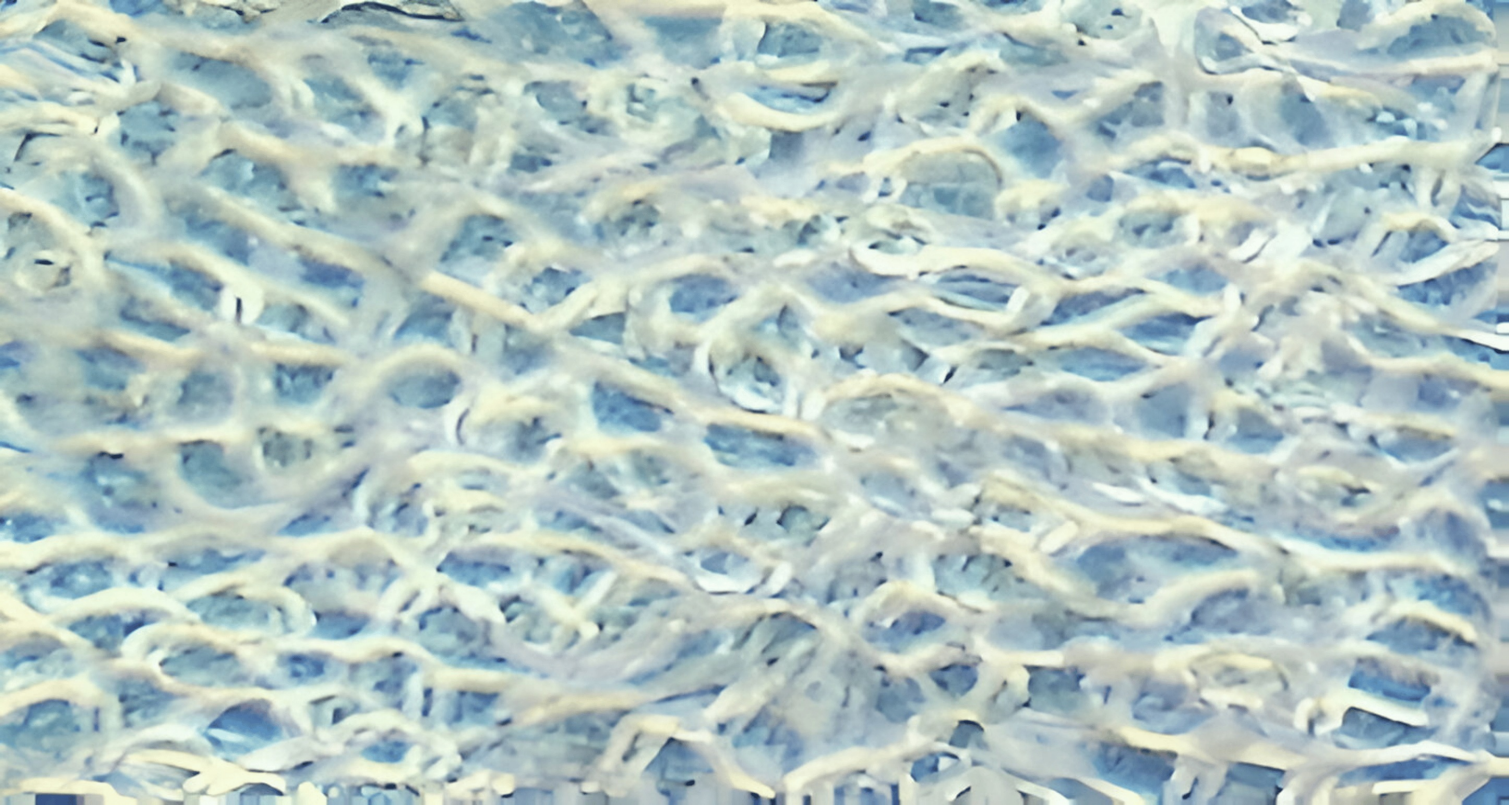
Human breast cancer cell line MDA-MB-231 used in the cytotoxicity study

In vitro, cytotoxicity trials were measured by means of MTT reduction. MDA-MB-231 cells (Figure 1) in a density of 6.5 × 106 cells per mL measured with an automated cell counter (Bio-Rad, France) were seeded in 96-well plates for 24 hour at 37 °C using an incubator (Memmert, Germany). The cells were sub-cultured in media (DMEM) comprising various concentrations of BSA NPs (0-500 μg/mL) under sterile conditions using a laminar flow hood (Labconco, USA). After 24 hours, the DMEM medium was changed with a fresh medium loaded with 10% MTT (5 mg/mL), and then the plates were incubated for additional four hours at 37 °C for the cytotoxicity study. Then, the medium was removed, with the addition of 100 μL DMSO to every well. Absorbance at 560 nm was measured using a multimode detector (Glomax, USA), and cell viability was computed using the following equation [19,20]:

Cell viability (%) = [ABS (Treated cells)] / [ABS (Control cells)] x 100

Statistical analysis

The study utilized one-way ANOVA to compare result parameters, determining statistically significant differences between groups at p values of P < 0.05 and non-significant at P > 0.05 using Microsoft Excel (Microsoft® Corp., Redmond, WA).

## Results

Impact of organic solvents on the physicochemical properties of BSA NPs

**Figure 2:**
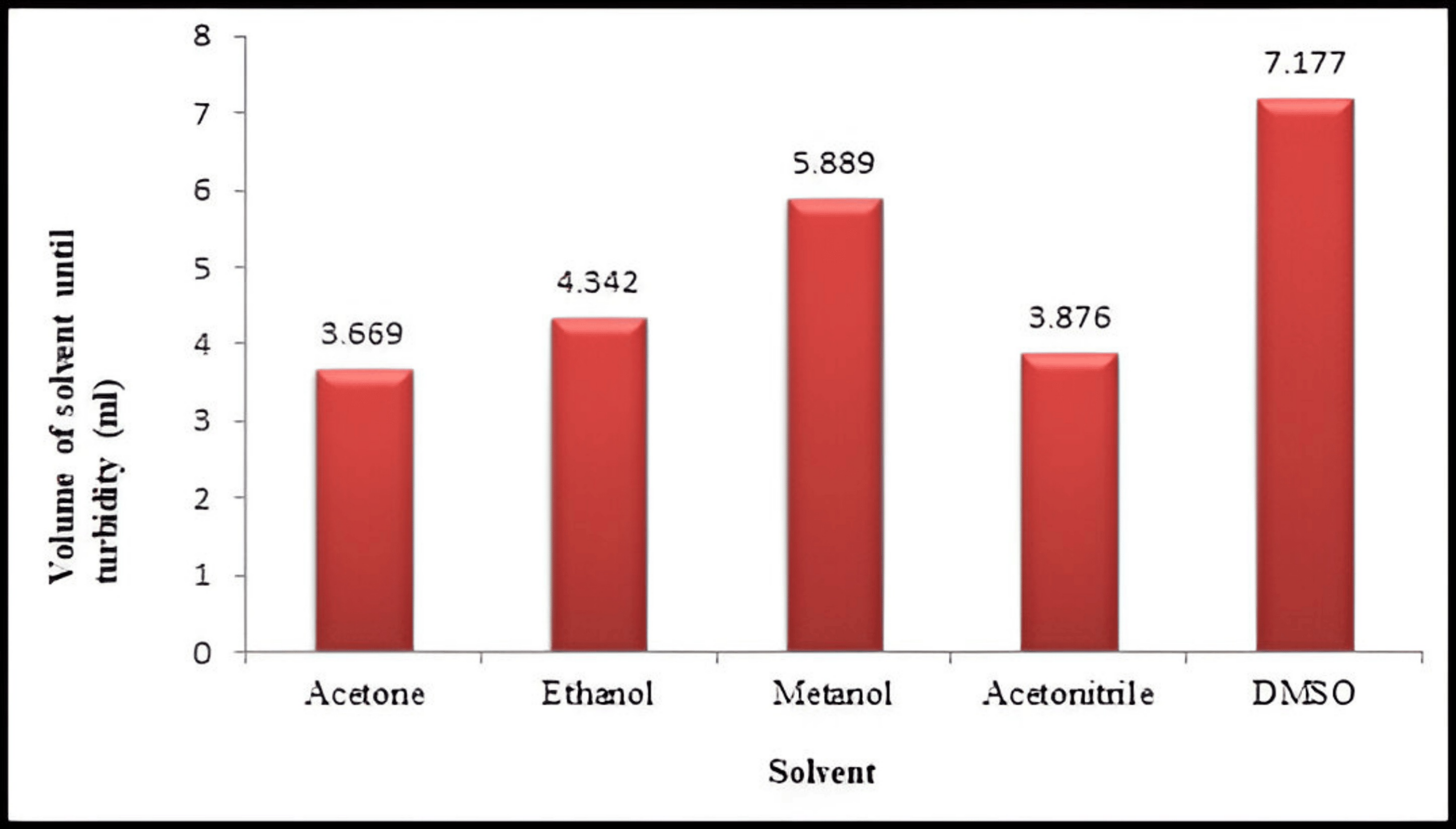
The volume of each organic solvent added to 5 mL of protein solution till it becomes turbid

The quantities of organic solvents are presented in Figure 2. Organic solvents were incorporated into the solution of BSA using a syringe pump under stirring at 1,250 rpm at room temperature (25 °C) till the solution became just turbid indicating NPs’ formation Extra addition of the organic solvent caused an increase in the density of particles. The results exhibited the ability of all the organic solvents to achieve NP albumin desolvation.

**Figure 3:**
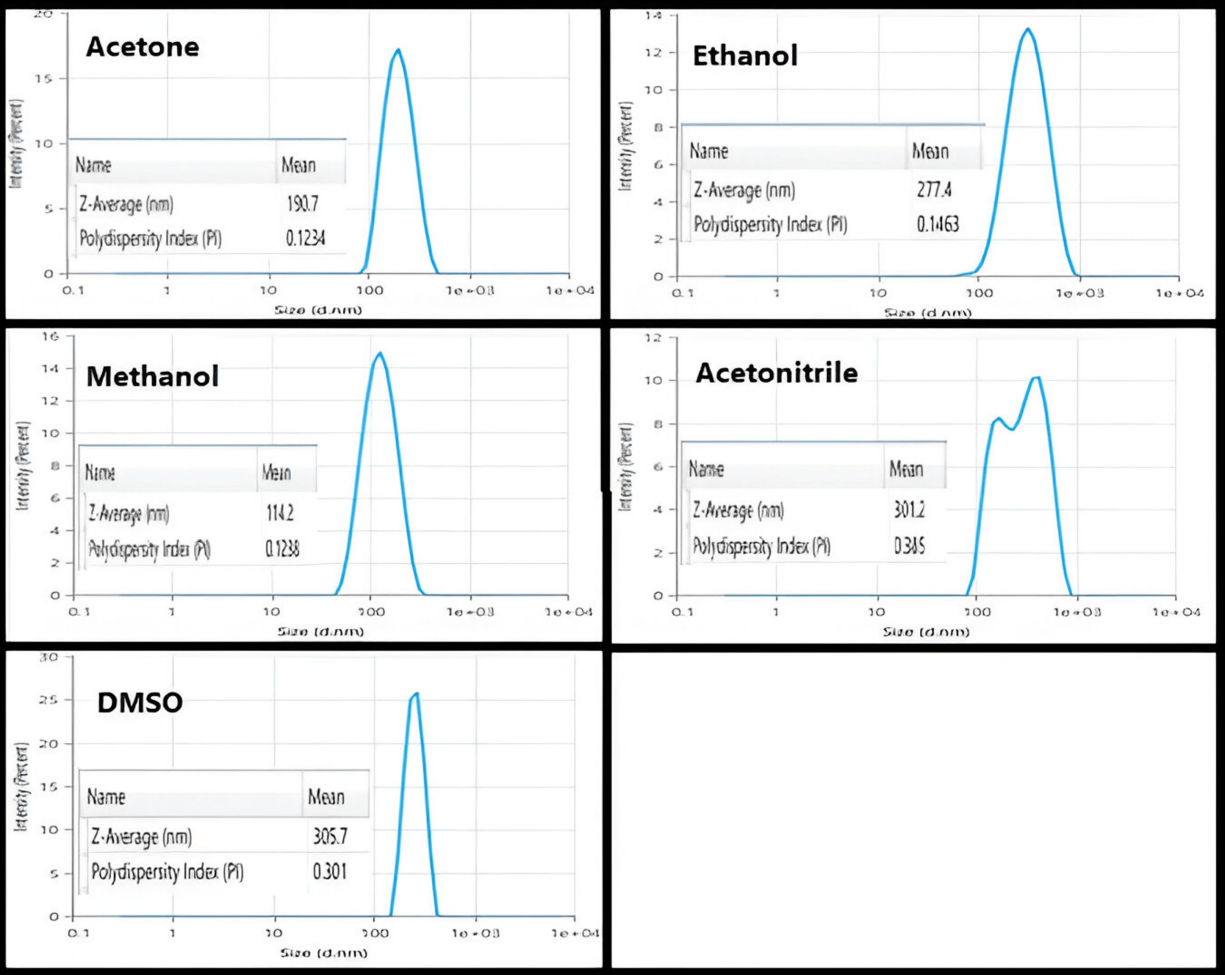
The particle size and size distribution of BSA NPs prepared by using different organic solvents

Figure 3 shows the particle size and size distribution of BSA NPs synthesised with several organic solvents. Methanol consumption caused BSA NPs (114.2 nm) to shrink in size. Moreover, a lot of additional solvent is required to produce the protein solution's turbidity (5.889 mL). Their minor opalescence could be related to the reduced size of BSA NPs produced by methanol. Polar aprotic solvents, acetone, and acetonitrile have smaller and bigger dielectric constants than ethanol, respectively. Using acetone produced the NPs with a 190.7 nm diameter, which were smaller than those synthesised with ethanol (277.4 nm). Less acetone than ethanol (3.669 mL against 4.342 mL) was required to produce turbidity in the protein solution. Acetonitrile and DMSO with the biggest dielectric constant were used to prepare BSA NPs. Its dielectric constant led one to expect acetonitrile to produce the smallest BSA NPs. However, the findings revealed that BSA NPs produced from acetonitrile and DMSO possessed both the biggest size and size distribution. By the way, 3.876 mL of acetonitrile was required to produce turbidity in the protein solution [12].

**Figure 4:**
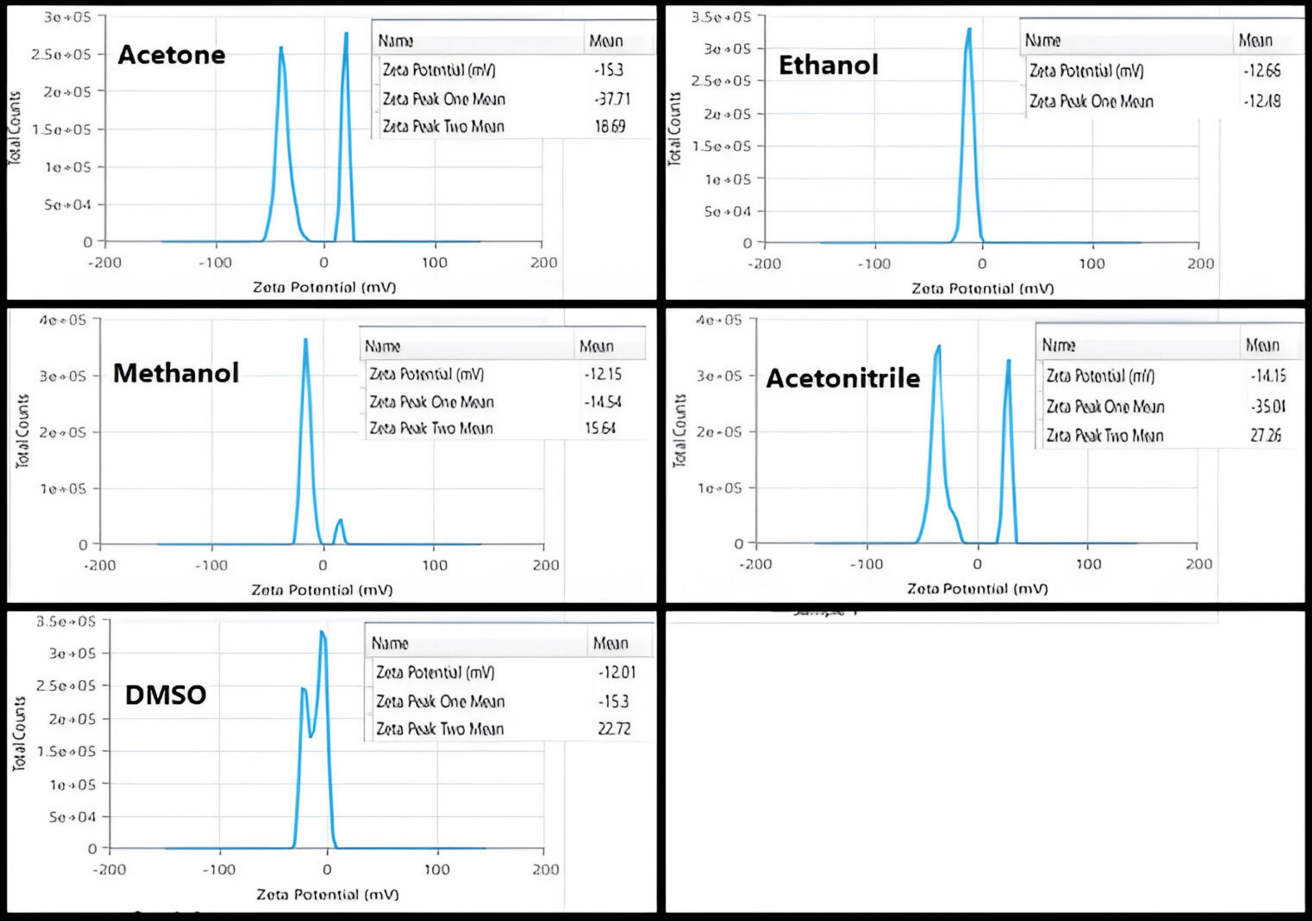
The zeta potential of BSA NPs prepared by using different desolvating agents

The kind of organic solvents had no important influence on the zeta potential of BSA NPs (Figure 4). The zeta potentials of the NPs were not highly affected by the organic solvents, varying between -12.01 and -15.30 mV [12].

Assessment of BSA structure after desolvation

**Figure 5:**
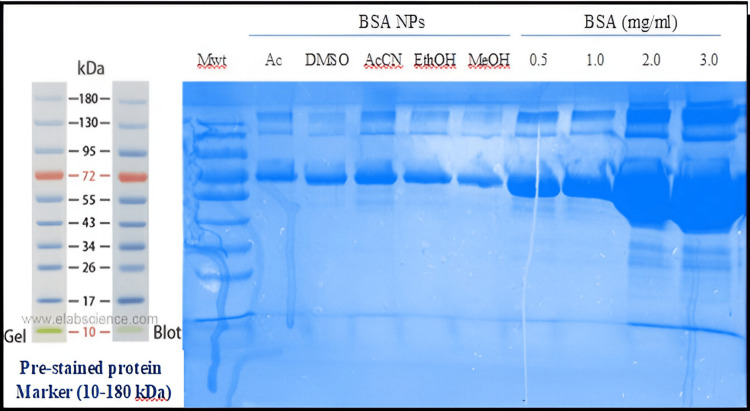
SDS-PAGE, 10 µL of samples (350 µg/mL) was loaded into a 10% polyacrylamide gel Mwt: molecular weight marker, lane one: acetone, lane two: DMSO, lane three: acetonitrile, lane four: ethanol, lane five: methanol

The organic solvents may cause fragmentation of BSA, so the impact of these solvents on BSA structure was evaluated by PAGE. A sharp band was detected near 72 kDa for untreated BSA and BSA after organic solvent treatments (Figure 5), suggesting that denaturation and fragmentation in BSA can be considered negligible in the desolvating process by the different organic solvents [21].

**Figure 6:**
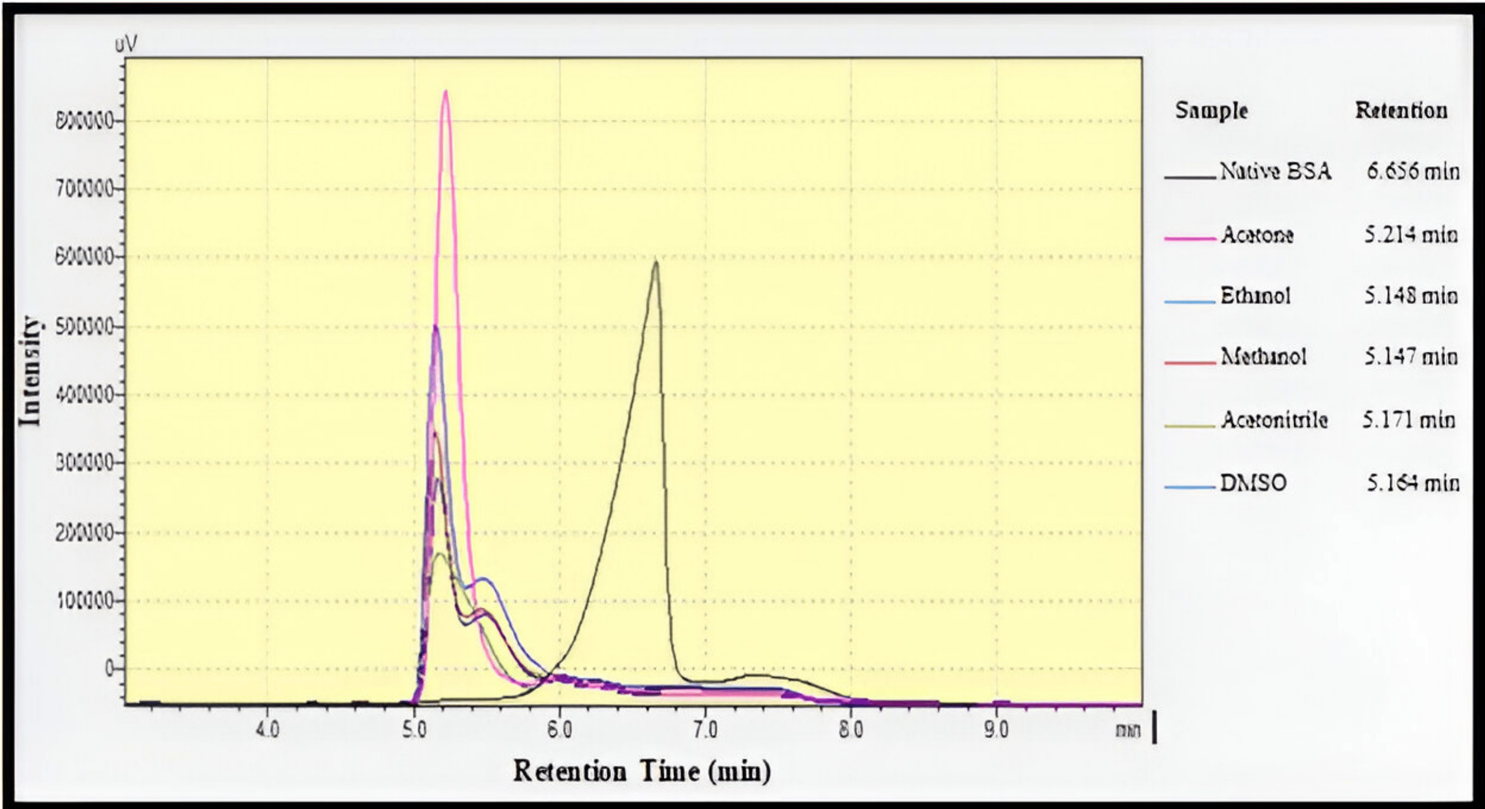
SEC-HPLC chromatograms of native BSA and BSA NPs prepared with different organic solvents

The result of SEC regarding organic solvents shows that the retention time of native albumin was 6.6 min. These results are in agreement with data previously reported [22,23]. The results of size exclusion chromatography are shown in Figure 6.

Human blood compatibility

Hemolysis Test

**Figure 7:**
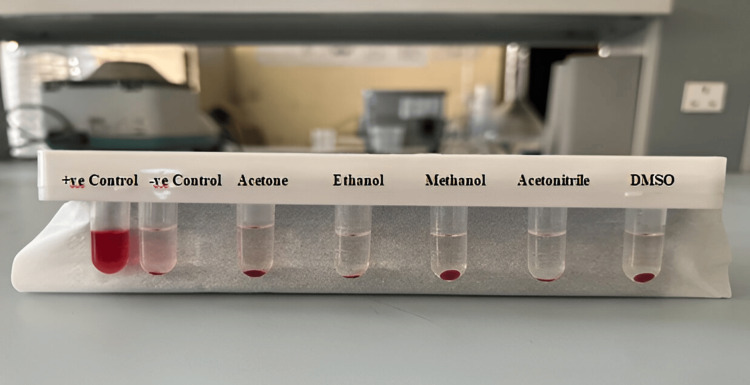
Hemolytic activity photographs of HRBCs treated with BSA NPs synthesized using different desolvating agents

BSA NP hemocompatibility can be verified through an assay of in vitro cytotoxicity to determine the erythrocyte biosafety. BSA NPs, as shown in Figure 7, showed strong hemocompatibility, close to PBS (negative control). In contrast, obvious hemolytic activity was seen in Triton X 100 (2% v/v) (positive control) treated RBC. Hemolytic activity percentages were less than 5% for BSA NPs (standard acceptance limit), indicating their excellent hemocompatibility [24].

Erythrocyte Aggregation Test

**Figure 8:**
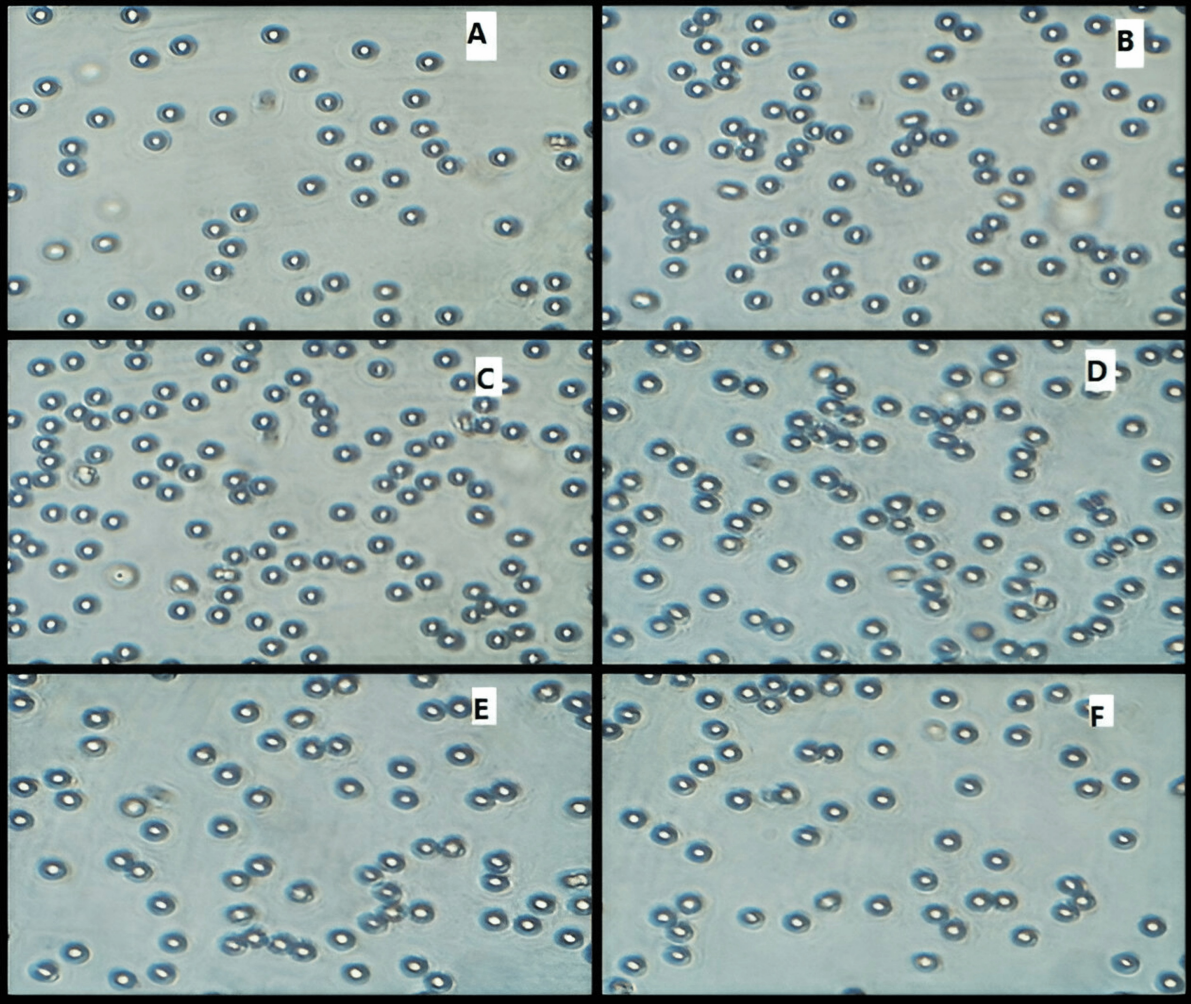
Light microphotograph of RBC aggregation (A) control, (B) acetone, (C) ethanol, (D) methanol, (E) acetonitrile, and (F) DMSO

As illustrated in Figure 8, fresh RBCs were circular in shape. After incubation with BSA NPs prepared with the different organic solvents in PBS, pH 7.4 for one hour at 37 °C, no aggregation of RBCs was reported. This showed that BSA NPs formulation was safe for erythrocytes [25].

Cell Viability Study

**Figure 9:**
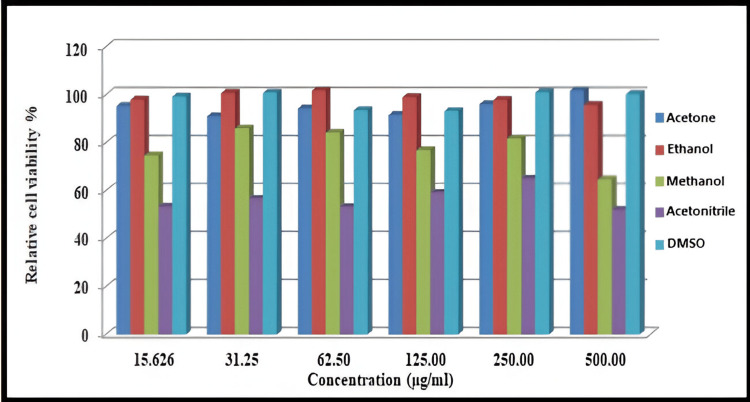
Cell viability assay of BSA NPs prepared with different organic solvents in MDA-MB-231 cells after 24 hours

In order to assess the cytotoxic effect of all preparations of BSA NPs on MDA-MB-231 cells, the in vitro cytotoxicity was studied using an MTT assay. It has been previously shown that the desolvating agents have toxicity on cells. MDA-MB-231 cells were incubated with serial concentrations of BSA NPs (15.625-500 μg/mL) [12]. The results of the cytotoxicity study are shown in Figure 9.

## Discussion

The chief aim of this study was to assess the impact of organic solvents with various dielectric constants, polarities, hydrogen bonding potentials, and functional groups on the particle size and polydispersity of BSA NPs and the possibility of using these NPs for delivery of drugs intravenously. Reducing the protein hydration resulted in raising the concentration of water-miscible solvents. Furthermore, reducing the dielectric constant of the aqueous mixture enlarged the size of the NPs. Therefore, smaller particle sizes resulted from desolvating substances with increased dielectric constant [26]. An increase in turbidity indicates that the density of particles increases without an increase in size. Methanol can disrupt the external hydrophilic layer in protein. The δp and δH of methanol are larger than ethanol, and its δ value is closer to that of water. Ethanol is more active than methanol in the solubilization of nonpolar groups since ethanol disrupts the protein structure with lesser volumes. Acetone with a lower value dielectric constant than that of ethanol produces BSA NPs with smaller sizes than those prepared with ethanol. The capacity of ethanol to cause disruption to the external layer of protein is greater as compared to acetone (depending on its δp and δH). Acetone is more able to interact with the non-polar areas of proteins. The lesser absolute concentration of acetone in the solution caused the dielectric constant of the acetone-protein mixture to be greater (59.86) even if acetone's dielectric constant was smaller than ethanol's. Hydrogen bonding enables the creation of bigger matrices and therefore large size of particles. Acetone is a hydrogen bond acceptor, while ethanol as a polar protic solvent is able to act as a hydrogen bond donor and acceptor. Less creation of hydrogen bonds by acetone allows the formation of smaller BSA NPs than those prepared by ethanol [12]. NPs synthesized with acetone were generally smaller and had a lower polydispersity index compared to those prepared with ethanol [27]. Acetonitrile can cause protein denaturation by changing the tertiary structure via hydrophobic interaction. Because of its high dielectric constant, it was predicted to produce NPs with small particle sizes. BSA NPs with a size of 301.2 nm were synthesized using acetonitrile with the lowest quantity to get a turbid solution. The value of acetonitrile has the lowest value of δH than other solvents [28]. This is so because the application of desolvating chemicals caused a significant decrease in the α-helical structure of the protein. Methanol produced BSA NPs smaller than those made by acetonitrile and DMSO. Furthermore, the α-helical structure was somewhat less than in the case using acetonitrile and DMSO [29,30]. Protein aggregation can boost biotherapeutics' immunogenicity and is a major contributor to negative events linked to immunogenicity in the clinic. Aggregation is a wide term, including the interactions that end in the self-association of molecules of protein into assemblies other than the natural quaternary structure [31]. Although there was a difference in the average retention times of all the prepared BSA NPs, which were 5.14 (lower than the native BSA), it did not present an important difference from the control (native protein), precluding that these samples are relatively native-like (i.e., denaturation and fragmentation in albumin appear to be insignificant in the desolvation process by different desolvating agents as evidenced by the absence of the new peaks). The SDS-PAGE analysis supported this evidence as described previously. The explanation for shifting the retention times of BSA NPs to the lower values as compared to the native BSA was related to many reasons. First, the SEC principle separates molecules based on their size in solution. Larger particles (here, different BSA NPs with particle sizes between (114-305 nm) are excluded from entering the pores of the stationary phase, leading to shorter retention times. NPs, depending on their size and structure, may be larger than native albumin, resulting in quicker elution [13]. The particle size of the native BSA is approximately 7 nm [32], so its elution was delayed.

Second, native BSA may have different interactions with the stationary phase compared to NPs because it remains in the unfolded state, which could also influence retention time. These interactions include electrostatic and hydrophobic bindings with stationary phase [33,34]. Meanwhile, for NPs, it undergoes many folded interactions, both inter- and intramolecular interactions as a result of the crosslinking effects, so they elute rapidly as compared to the native form. The albumin NPs prepared by acetone, ethanol, and DMSO showed more than 90% cell viability and non-significant cytotoxicity in MDA-MB-231 as compared to the control, which showed 100% cell viability regarding all the six concentrations used in the MTT assay, indicating the possible use of these systems to deliver drugs [9]. On the other hand, the albumin NPs prepared by methanol and acetonitrile showed a significant cytotoxicity effect in MDA-MB-23 cells as compared to control for all six concentrations used in the MTT assay as these solvents refer to class 2, which are associated with fewer severe toxicity and must be limited to protect patients from possible adverse effects [35].

Limitations of the study

One limitation of this study is the lack of in vivo experimentation to validate the potential therapeutic efficacy of the synthesized glutathione-cross-linked BSA NPs for drug delivery intravenously. While the study extensively examines the physical and chemical properties of the NPs and their compatibility with blood components, the absence of in vivo studies limits the understanding of how these NPs would behave in a living system. Without data on factors such as circulation time, tissue distribution, and pharmacokinetics in a biological environment, the practical utility of these NPs as drug carriers remains speculative. Additionally, the cytotoxicity assessments were mainly conducted on MDA-MB-231 cells in vitro, which may not fully represent the complexities of interactions with different cell types or physiological systems in vivo. This gap in research will be considered in the second part of the study.

## Conclusions

The desolvation technique is a fast and easy technique in the synthesis of protein NPs. In this study, the preparation conditions of BSA NPs were optimized with suitable size by the desolvation method. The use of different desolvating agents in the desolvation process resulted in the formation of NPs with a variety of properties. The smallest particles can be obtained with the use of methanol and then acetone, whereas the use of DMSO produced the largest particles. The results of SDS-PAGE and SEC suggest that denaturation, fragmentation, and aggregation in BSA appear to be negligible in the desolvating process as evidenced by the absence of new peaks. The steps of this method are simple. The resulting cross-linked BSA NPs own good blood compatibility. As no significant cytotoxicity of the NPs was detected, except for those prepared using methanol and acetonitrile, it can be decided that the BSA NPs of serial concentrations have no toxicity, and the method of eliminating organic solvent and extra glutathione is excellent, proving that these NPs are biocompatible. Therefore, acetone was found to be a suitable desolvating solvent for addition to the BSA solution, followed by the addition of glutathione as a cross-linker. As the particles with size (100-200 nm) accumulate at the cancer and site of inflammation after administration intravenously, the cross-linked BSA NPs synthesized by the desolvation method can be a new nanocarrier for the intravenous delivery of anticancer drugs.
